# Multiple Neurological Complications and Multidrug-Resistant Bacterial Infections During Prolonged Intensive Care for Undocumented Meningoencephalitis in Côte d'Ivoire: A Case Report

**DOI:** 10.7759/cureus.117752

**Published:** 2026-10-11

**Authors:** Setondji Emmanuel Raymond Ahouangansi, Loes Koffi, Zolé Cédric Doh, Kouame Koffi Isidore, Walamitien Cyrille Touré, Lossan Herman Kra, Stephane Charles Evrard Adingra, Floriane Mouafo, Aichata Bamba-Traore, Yves Gontran Lobah, Pindjou Kpata Djami

**Affiliations:** 1 Anesthesia and Critical Care, Université Félix Houphouët-Boigny, Abidjan, CIV; 2 Geriatrics, Université Félix Houphouët-Boigny, Abidjan, CIV; 3 Internal Medicine, Université Félix Houphouët-Boigny, Abidjan, CIV

**Keywords:** central diabetes insipidus, côte d'ivoire, hydrocephalus, medical intensive care unit (micu), spinal cord ischemia

## Abstract

Acute meningoencephalitis requiring intensive care may be complicated by severe neurological injury and nosocomial infections. Hydrocephalus is well recognized, whereas spinal cord ischemia, central diabetes insipidus, and flaccid tetraparesis are rarely reported. Their coexistence, together with successive infections involving multidrug-resistant organisms, presents substantial diagnostic and therapeutic challenges.

A previously healthy 32-year-old man presented with neck and back pain following a road traffic accident, subsequently developing cerebellar signs, acute confusion, and meningeal signs without fever. Cerebrospinal fluid (CSF) analysis revealed neutrophilic pleocytosis, elevated protein, and marked hypoglycorrhachia. Microbiological investigations were negative, and no causative pathogen was identified. Empirical antimicrobial therapy was followed by normalization of the CSF leukocyte count by hospital day 6. Despite this initial response, the patient required mechanical ventilation and developed shunt-dependent tetraventricular hydrocephalus, probable central diabetes insipidus, imaging findings suggestive of thoracic spinal cord ischemia, and areflexic flaccid tetraparesis with diffuse axonal sensorimotor neuropathy. His prolonged intensive care course was further complicated by successive nosocomial infections involving multidrug-resistant bacteria. He died on hospital day 70 from septic shock associated with *Enterobacter cloacae* bacteremia and multiorgan failure.

Negative microbiological findings do not exclude meningoencephalitis when clinical and CSF abnormalities support the diagnosis, although alternative etiologies require consideration. Improvement in CSF findings does not preclude subsequent neurological deterioration or nosocomial infection. Continued neurological assessment, rigorous infection-prevention measures, and prompt investigation of suspected nosocomial infections remain essential throughout prolonged intensive care, particularly in resource-limited settings.

## Introduction

Acute bacterial meningoencephalitis remains associated with mortality exceeding 30% among affected adults, even though most causative bacteria remain susceptible to available antibiotics [1]. This apparent discrepancy reflects the pathophysiology of the disease: neurological injury results not only from bacterial invasion but also from the inflammatory response within the subarachnoid space, which may persist after bacterial clearance. In Côte d'Ivoire, this disease remains poorly characterized in adults. The only available hospital-based surveillance focused on pediatric patients and identified a bacterial cause in just 3% of suspected meningitis cases recorded between 2010 and 2016 [2].

Two challenges complicate initial management. The first is recognizing the disease: clinical features cannot reliably distinguish bacterial meningitis from other infectious and non-infectious conditions. Insidious presentations without fever or laboratory evidence of systemic inflammation are particularly misleading when a concurrent event offers a plausible alternative explanation for the symptoms [1]. The second is identifying the causative organism: the sensitivity of Gram staining and culture falls below 50% when patients have received antibiotics, even an incomplete course, before cerebrospinal fluid (CSF) collection [3].

Prognosis is influenced by two distinct issues that are rarely considered together in the literature. The first is neurological complications. Acute hydrocephalus is well recognized and associated with a poor prognosis, whereas spinal cord ischemia, central diabetes insipidus, and post-infectious polyradiculoneuropathy have been described only in isolated reports [4-7]. The second is nosocomial infection with multidrug-resistant organisms, which is an independent predictor of poor outcomes in intensive care and is associated with increased mortality and prolonged hospitalization [8,9]. Both types of complications can progress despite initial antibiotic therapy and microbiological control of the meningeal infection.

We report the case of a 32-year-old man diagnosed with meningoencephalitis based on clinical and CSF findings, despite the absence of an identified pathogen following a comprehensive diagnostic workup performed before antibiotic administration. Although his CSF normalized within six days, he developed four successive neurological complications and multiple bacterial and fungal infections, including infections caused by multidrug-resistant bacteria. After 70 days in intensive care, he died of septic shock. The rationale for reporting this case is to highlight the coexistence of multiple uncommon neurological complications and successive nosocomial infections while illustrating the diagnostic uncertainty and management challenges encountered during prolonged intensive care in a resource-limited setting. It also emphasizes that improvement in the initial CSF findings does not necessarily indicate a favorable subsequent clinical course.

## Case presentation

Initial presentation and clinical assessment

A 32-year-old man with no known medical or surgical history presented with severe neck and back pain of two weeks' duration following a road traffic accident. He had been seated in the rear of the vehicle and reported experiencing a sensation of spinal compression when the vehicle crossed a speed bump. Two days later, he developed severe headache, tinnitus, and positional dizziness, accompanied by marked fatigue and unquantified weight loss. Because the pain persisted despite self-treatment with paracetamol, he sought medical care and was hospitalized. 

On initial examination, the patient appeared unwell. His blood pressure was 179/111 mmHg, his heart rate was 100 beats/min, his oxygen saturation was 97% on room air, his temperature was 37.2°C, and his Glasgow Coma Scale score was 15 [10]. Neck movement elicited pain, and examination revealed muscle spasm and brisk deep tendon reflexes; however, he had no sensorimotor deficits or meningeal signs. Brain magnetic resonance imaging (MRI) showed early leukoaraiosis but no cerebellar lesions (Figure 1).

**Figure 1:**
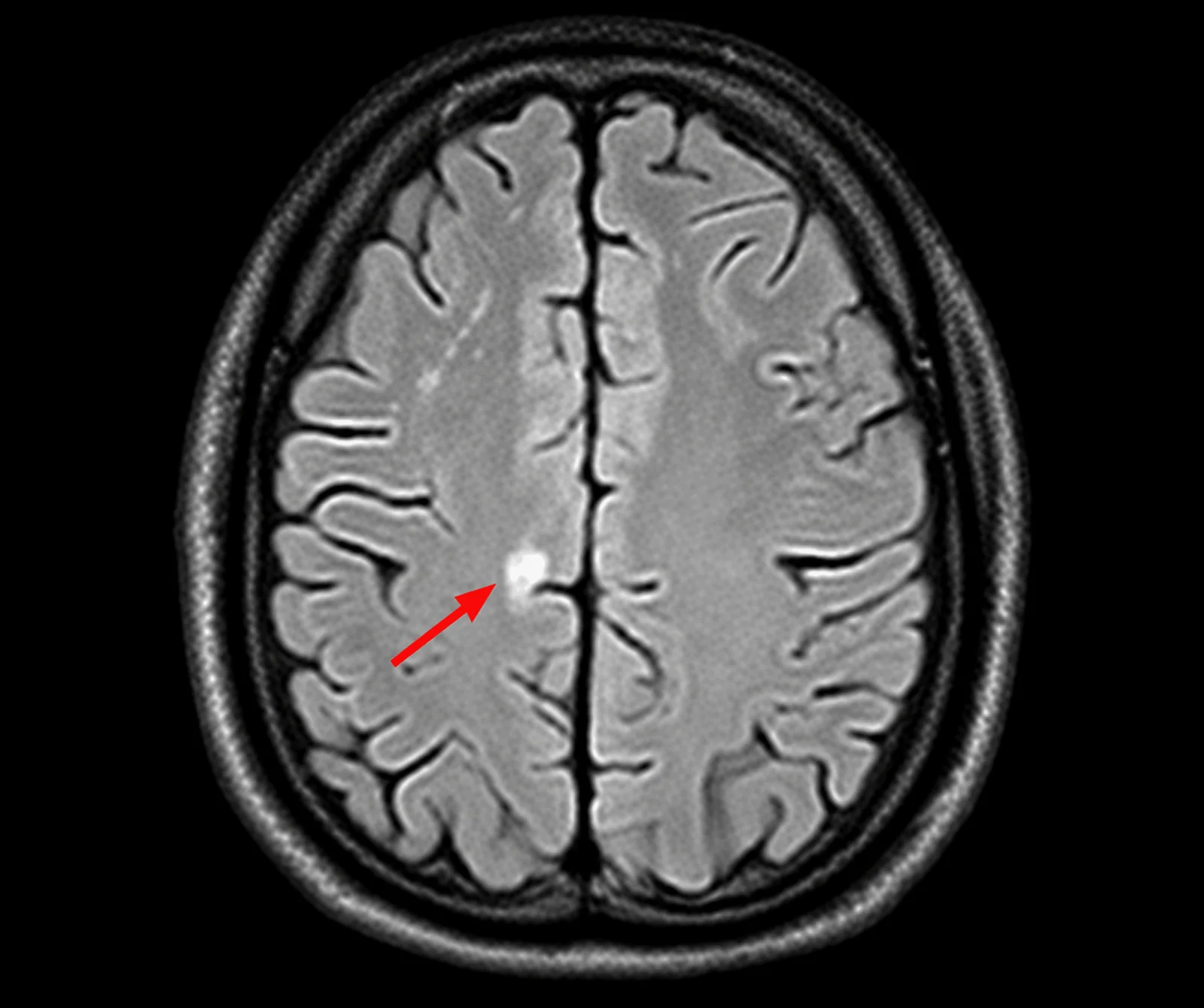
Brain magnetic resonance imaging demonstrating leukoaraiosis

Computed tomography (CT) of the cervical and lumbar spine showed C5-C6 uncovertebral arthrosis and multilevel disc protrusions causing dural and nerve root impingement (Figure 2).

**Figure 2:**
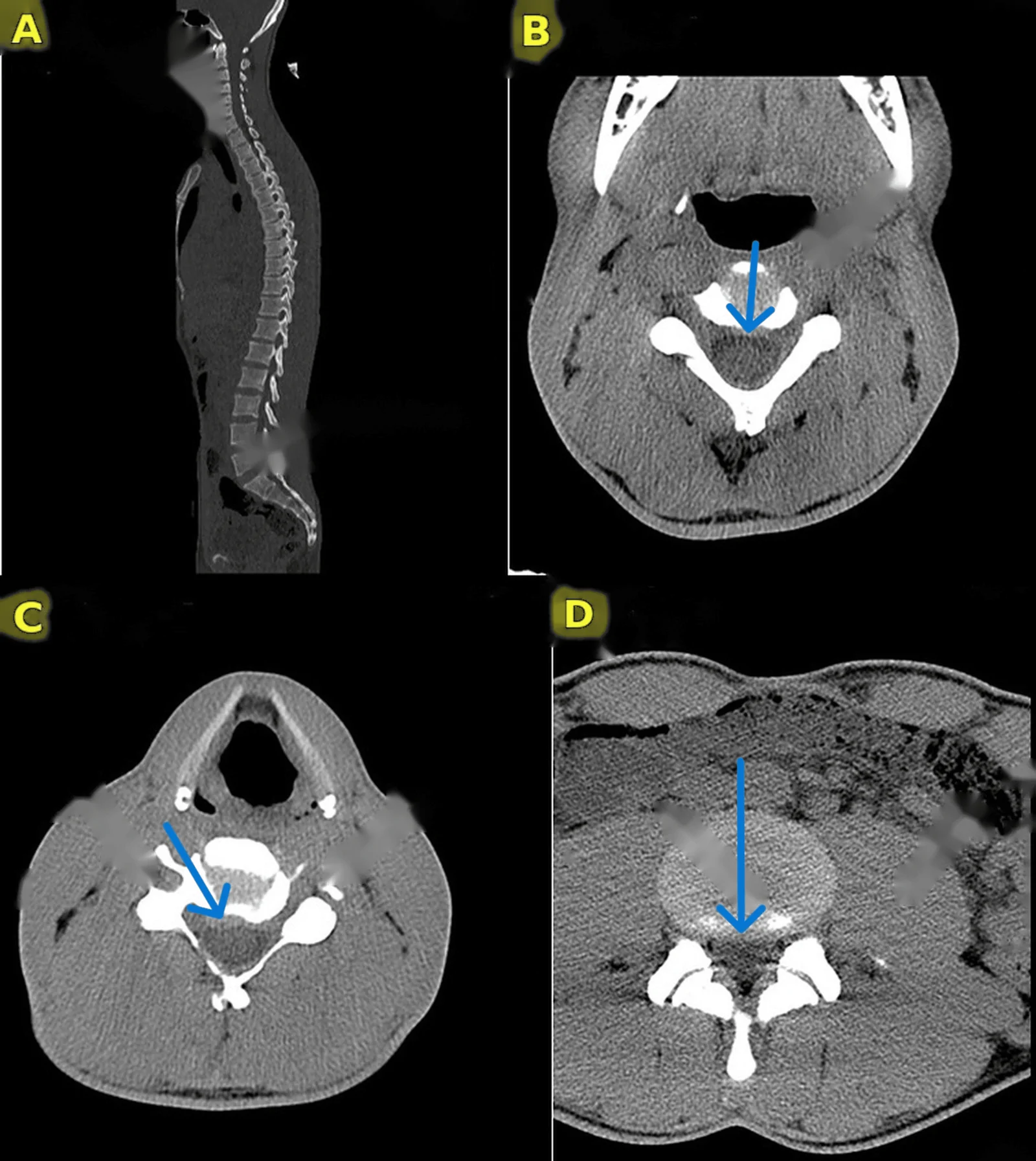
Non-contrast computed tomography scan of the entire spine, acquired in axial sections (B, C, and D) with sagittal reconstruction (A), demonstrating multilevel degenerative disc disease. On the bone window, the normal physiological spinal curvatures are preserved (A). There are a posterior median disc bulge at C2-C3 with dural sac impingement (B), a right posterolateral disc protrusion at C4-C5 with dural sac impingement (C), and a broad-based disc bulge at L4-L5 with dural sac impingement (D)

Neurological deterioration and diagnostic evaluation

On hospital day 1, the patient developed a cerebellar syndrome characterized by ataxia, dysarthria, and an unsteady gait, without motor or sensory deficits. Imaging showed no cerebellar lesions. On days 2 and 3, he developed acute confusion followed by overt meningeal signs. Laboratory testing showed a peripheral white blood cell count of 22,160/mm³ (71% neutrophils), a C-reactive protein (CRP) level of 1.51 mg/L, and a procalcitonin level of 0.69 ng/mL. The marked leukocytosis contrasted with the relatively low levels of the other inflammatory markers.

The CSF was turbid and contained 139 leukocytes/mm³, with neutrophil predominance; the corrected leukocyte count after accounting for a traumatic tap was 131 cells/mm³. The CSF protein concentration was 2.57 g/L, and the glucose concentration was 0.11 g/L (Table 1). Gram staining, bacterial and fungal cultures, soluble antigen testing for *Neisseria meningitidis *serogroups A, B, and C, *Haemophilus influenzae*, *Streptococcus pneumoniae*, *Streptococcus agalactiae*, and *Cryptococcus neoformans*, and a syndromic multiplex polymerase chain reaction (PCR) panel performed on the same sample were all negative (Tables 1-2).

**Table 1:** Evolution of cerebrospinal fluid findings

Parameter	Unit	Reference range	Day 3	Day 6	Day 9
Appearance	-	Clear	Slightly turbid	Clear	Clear
White blood cells	cells/mm³	<5	139 (71% neutrophils)	<2	<2
Red blood cells	cells/mm³	0	>2,000	<10	164
Protein	g/L	0.10-0.30	2.57	0.81	0.40
Glucose	g/L	0.40-0.70	0.11	0.07	0.70
Chloride	mmol/L	120-130	88	Not performed	104
Gram stain	-	Negative	Negative	Negative	Negative
Bacterial/fungal cultures	-	Negative	Negative	Negative	Negative
Molecular panel	-	Negative	Not performed	Negative	Not performed

**Table 2:** Timeline of clinical, laboratory, and therapeutic events MRI: magnetic resonance imaging; CSF: cerebrospinal fluid; EMG: electromyography; PCT: procalcitonin

Day	Clinical event	Investigations and treatment
Day 1	Isolated kinetic cerebellar syndrome	Brain MRI: leukoaraiosis. C5-C6 cervical arthrosis
Day 2	Acute confusional state	Light sedation
Day 3	Overt meningeal syndrome	White blood cell count 22,160/mm³, CRP 1.51 mg/L. Abnormal CSF, negative microbiology. Ceftriaxone, gentamicin, aciclovir
Day 6	Afebrile, meningeal signs resolving	CSF cytologically normalized. Negative CSF molecular panel
Day 7	Coma, acute respiratory failure	ICU admission, intubation. Iatrogenic pneumothorax drained. Cefepime, vancomycin
Day 8	Tetraventricular hydrocephalus	External ventricular drain with pressure sensor
Days 8-11	Polyuria, hypernatremia up to 156 mmol/L	Desmopressin
Day 9	Respiratory colonization	Positive respiratory panel: *Acinetobacter *and *Klebsiella*, 10⁷ copies
Days 8-15	Repeated clamping failures	Second external ventricular drain
Days 16-68	Persistent shunt dependence, right mydriasis	Ventriculoperitoneal shunt, variable-pressure valve
Days 16-68	Areflexic flaccid tetraparesis after sedation discontinued	Axonal EMG. Spinal MRI. Immunoglobulin 2 g/kg and methylprednisolone
Days 16-68	Failure to wean from ventilation	Surgical tracheostomy, peristomal bleeding
Day 69	Septic shock	Respiratory panel positive for six organisms. Blood culture: *Enterobacter cloacae*. PCT 295 ng/mL
Day 70	Refractory cardiac arrest	Death

The sample had been collected before the administration of any antibiotics in the hospital. Human immunodeficiency virus (HIV) and syphilis serologic test results were also negative. Urinalysis revealed marked sterile pyuria, raising the possibility of a partially treated infection or an infection caused by intracellular organisms. 

Initial treatment and early response

Empirical antimicrobial therapy with ceftriaxone (2 g twice daily), gentamicin (160 mg/day), and aciclovir (500 mg every eight hours), together with adjuvant dexamethasone, was initiated on the day of diagnosis. By day 6, the leukocyte count in a follow-up CSF sample had normalized (<2 cells/mm³), the protein concentration had decreased to 0.81 g/L, and repeat testing with the molecular panel remained negative (Table 3).

**Table 3:** Microbiological samples Loads expressed as powers of 10 correspond to DNA copy numbers (molecular panels), not colony-forming units. Susceptibility testing was performed on the VITEK 2 automated system (bioMérieux, Marcy-l'Étoile, France). Minimum inhibitory concentrations were not available. S: susceptible; I: susceptible, increased exposure; R: resistant

Sample	Technique	Result
Cerebrospinal fluid, day 3	Gram stain, cultures, soluble antigens	Negative. *Cryptococcus *negative
Urine, day 3	Urinalysis and culture	>200 leukocytes/mm³, negative cultures. Abacterial leukocyturia
Cerebrospinal fluid, day 6	Molecular panel	Negative, on a post-antibiotic, normalized sample
Respiratory, day 7	Molecular panel	Negative
Respiratory, day 9	Molecular panel	*Acinetobacter *10⁷, *Klebsiella *10⁷, *Escherichia coli *10⁵ copies
Sputum	Ziehl-Neelsen and GeneXpert MTB/RIF	Negative
Bronchial aspirate	Culture	*Klebsiella pneumoniae *(semi-quantitative). *Candida tropicalis*
Klebsiella pneumoniae	Susceptibility testing	S: imipenem, meropenem, amikacin, piperacillin-tazobactam, ceftazidime-avibactam, ceftolozane-tazobactam, colistin. I: cefepime. R: ceftazidime, aztreonam, levofloxacin, ciprofloxacin, tobramycin. CTX-M and mecA/mecC, MREJ detected
Candida tropicalis	Antifungal susceptibility	Susceptible to all six antifungals tested, including fluconazole
Blood culture	Culture	Sterile
Blood culture	Culture	*Staphylococcus haemolyticus *and then *Staphylococcus capitis*
Respiratory	Molecular panel	*Acinetobacter*, *Klebsiella*, *Streptococcus agalactiae *10⁷; *Escherichia coli *and *Staphylococcus aureus *10⁶. CTX-M and mecA/mecC, MREJ
Respiratory, day 69	Molecular panel	*Acinetobacter *and *Escherichia coli *≥10⁷; *Staphylococcus aureus* 10⁶; *Pseudomonas aeruginosa *10⁵; *Enterobacter cloacae *10⁴; *Klebsiella*. CTX-M and mecA/mecC and MREJ
Blood culture, day 69	Culture	*Enterobacter cloacae*, aerobic and anaerobic

Intensive care admission and neurological complications

On day 7, the patient experienced progressive neurological deterioration, with his Glasgow Coma Scale score decreasing from 10 to 8, and acute oxygen desaturation to 78%, prompting admission to the ICU. Bilateral pneumonia was accompanied by markedly elevated CRP (196.97 mg/L) and procalcitonin (13.60 ng/mL) levels, despite a declining peripheral white blood cell count. High-anion-gap metabolic acidosis was present without hyperlactatemia. Central venous catheter placement was complicated by an iatrogenic pneumothorax, which required drainage. The patient was intubated and placed under sedation and mechanical ventilation, and antimicrobial therapy was broadened to cefepime and vancomycin.

On the second day in the ICU, the patient developed tetraventricular hydrocephalus without cerebral edema. An external ventricular drain and intracranial pressure monitor were placed. After repeated unsuccessful drain-clamping trials and neurological deterioration with right pupillary dilation following drain placement, a ventriculoperitoneal shunt was placed. The valve setting was subsequently reduced from 200 to 110 mmH2O, resulting in the normalization of the pupil size and improved consciousness. After sedation was discontinued, areflexic flaccid weakness of all four limbs was observed. Electromyography demonstrated diffuse axonal sensorimotor neuropathy. MRI of the cervico-thoraco-lumbar spine showed thoracic spinal cord atrophy consistent with ischemic injury (Figure 3), cervical spinal cord edema suggestive of Wallerian degeneration (Figure 4), and lumbar paraspinal muscle atrophy consistent with denervation.

**Figure 3:**
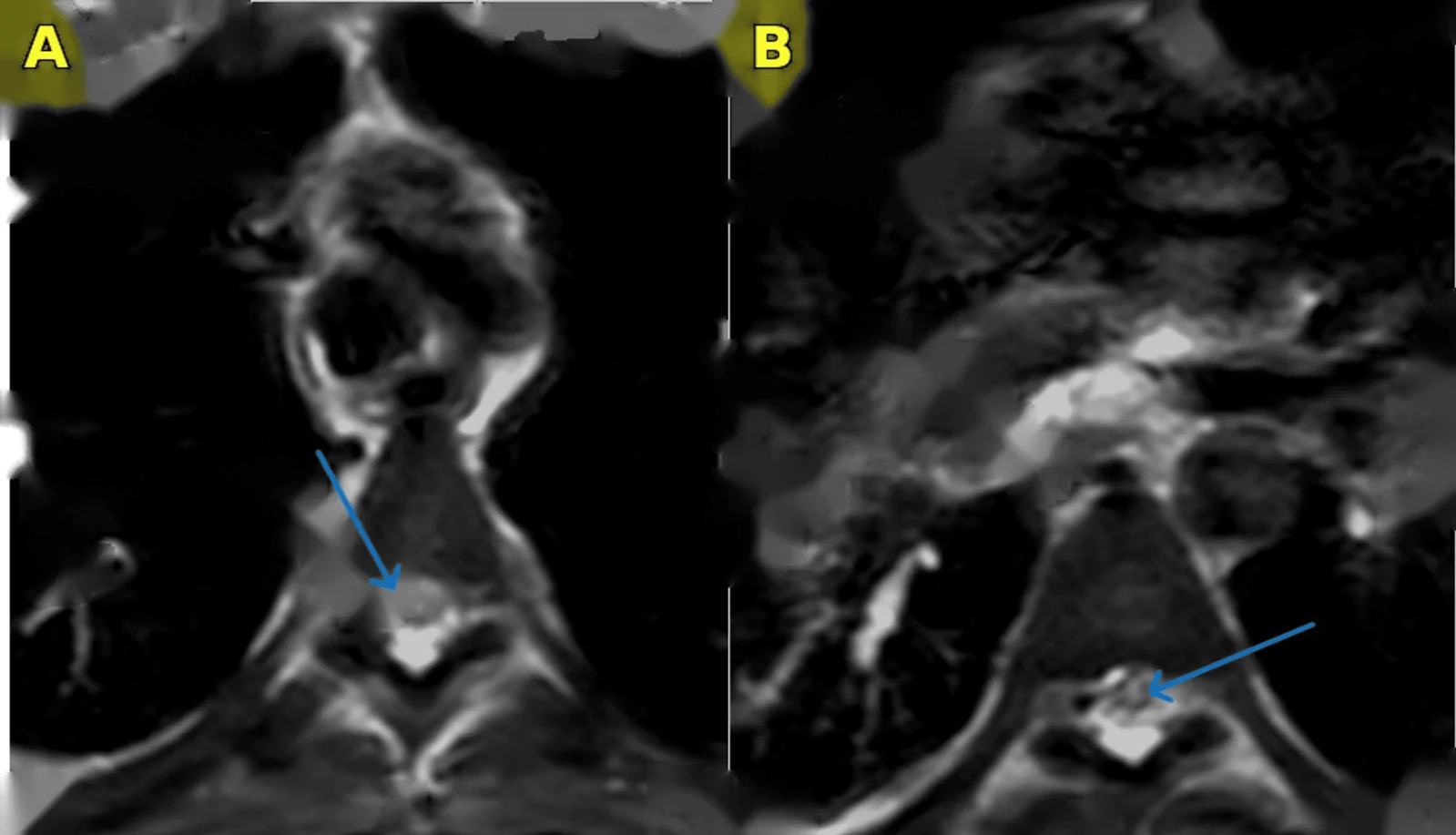
Axial T2-weighted sequences of the thoracic spine demonstrating focal atrophy of the thoracic spinal cord (A), associated with subtle intramedullary T2 hyperintensity of likely chronic sequelae, which may be compatible with prior spinal cord ischemia (B)

**Figure 4:**
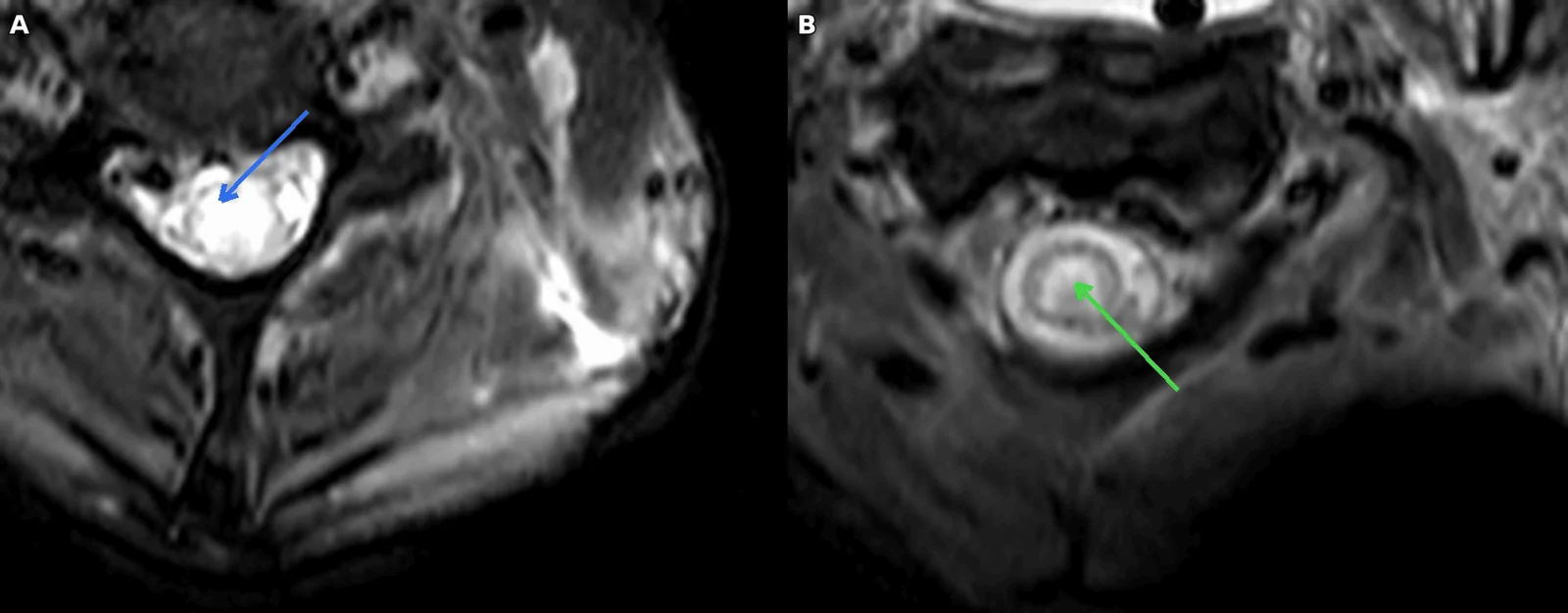
Axial T2-weighted sequences of the cervical spine demonstrating intramedullary T2 hyperintensity consistent with spinal cord edema, suggestive of Wallerian degeneration (A, B)

Acute post-infectious polyradiculoneuropathy was suspected and treated with intravenous immunoglobulin (2 g/kg) and methylprednisolone; however, no motor improvement was observed. Testing for anti-ganglioside and anti-myelin-associated-glycoprotein antibodies yielded negative results. CSF was not collected from the shunt at this stage to assess for albuminocytologic dissociation. Between ICU days 2 and 5, the patient developed hypotonic polyuria of 3-4 L/24 hours and progressive hypernatremia, with a peak sodium concentration of 156 mmol/L. The polyuria and hypernatremia responded to desmopressin, suggesting central diabetes insipidus; however, plasma and urine osmolalities were not measured.

Nosocomial infection and antimicrobial management

Nosocomial pathogens were detected rapidly after ICU admission (Table 3). A respiratory molecular panel that was negative on ICU day 2 detected high loads of *Acinetobacter calcoaceticus-baumannii* complex, *Klebsiella pneumoniae *group, and *Escherichia coli *48 hours later. Phenotypic antimicrobial susceptibility testing of the *K. pneumoniae* isolate, together with detection of a CTX-M gene, was consistent with extended-spectrum beta-lactamase production; the isolate remained susceptible to carbapenems. Concurrent detection of mecA/C and MREJ indicated the presence of methicillin-resistant *Staphylococcus aureus*. *Candida tropicalis* was repeatedly isolated from bronchial secretions and was susceptible in vitro to all antifungal agents tested, including fluconazole. Nevertheless, amphotericin B was administered because of the high prevalence of fluconazole resistance in the ICU at that time. Subsequent blood cultures grew vancomycin-susceptible *Staphylococcus haemolyticus *and, later, vancomycin-susceptible *Staphylococcus capitis*. During the terminal episode, the procalcitonin concentration increased to 295 ng/mL, the platelet count decreased to <30,000 cells/mm³, and the patient developed septic shock. A respiratory molecular panel detected *Enterobacter cloacae* complex, *Acinetobacter calcoaceticus-baumannii* complex, *E. coli*, *S. aureus*, *Pseudomonas aeruginosa*, and *K. pneumoniae*, as well as the CTX-M, mecA/C, and MREJ resistance markers. A concurrent peripheral blood culture grew *E. cloacae*. The final antimicrobial regimen consisted of meropenem, amikacin, vancomycin, and inhaled colistin.

Other intensive care complications

The respiratory course was complicated by recurrent bronchial secretions requiring repeated fiberoptic bronchoscopic aspiration and bronchoalveolar lavage. Because the patient could not be weaned from mechanical ventilation, a surgical tracheostomy was performed. The procedure was complicated by peristomal bleeding that required surgical hemostasis. Other complications included hypoalbuminemia (29 g/L; reference range: 40-50 g/L), cachexia, infected sacral pressure injuries, and anemia, with the hemoglobin concentration decreasing from 12.6 to 9 g/dL. The anemia occurred in the context of pre-existing, unexplained hypochromic microcytosis.

Outcome

On hospital day 70, the patient died of septic shock and multiorgan failure following cardiac arrest that was refractory to resuscitation.

## Discussion

This case is notable for two reasons. First, the patient developed four severe neurological complications: hydrocephalus, central diabetes insipidus, spinal cord ischemia, and suspected acute post-infectious polyradiculoneuropathy. Several of these complications are rarely reported following meningoencephalitis, and to our knowledge, their occurrence in a single patient has not previously been described. Second, the prolonged intensive care course was complicated by successive nosocomial bacterial and fungal infections, including infections caused by multidrug-resistant bacteria, culminating in fatal septic shock. The literature rarely addresses the combined prognostic burden of neurological complications and nosocomial infections, despite their particular relevance in resource-limited intensive care settings.

Initial diagnostic challenges

The classic triad of fever, meningeal signs, and altered consciousness was absent at presentation. The patient initially presented with a cerebellar syndrome and subsequently developed acute confusion and meningeal signs without fever or an elevated CRP level, despite markedly abnormal CSF findings. Moreover, the recent road traffic accident offered a plausible mechanical explanation for the initial spinal pain and headache, potentially delaying recognition of the infection. Ellis et al. noted that clinical features alone cannot reliably distinguish bacterial meningitis from other conditions, underscoring the importance of prompt diagnostic evaluation and empiric treatment when the disease is suspected [1]. The diagnostic workup of the initial CSF sample, which was collected before the administration of antibiotics in the hospital, yielded negative results on Gram staining, bacterial and fungal cultures, soluble antigen testing, and a syndromic molecular panel. In the study by Chahid et al., this panel demonstrated 100% sensitivity and a 100% negative predictive value for the pathogens included in the assay, but a positive predictive value of only 31% [11]. These findings are limited by the panel's restricted pathogen spectrum. In addition, undocumented antibiotic use before admission could have reduced the sensitivity of Gram staining and culture to below 50% [12], although prior antibiotic exposure would be less likely to account for a negative molecular test [3]. Nonprescription antibiotic use is an important cause of low culture yields in settings where this practice is common [12]. Although such exposure was possible in this patient, it could not be verified retrospectively. Neuroborreliosis was considered unlikely because serum serologic testing performed approximately six weeks after symptom onset yielded negative results, reducing the likelihood of a false-negative result attributable to testing during the early serologic window.

Neurological complications

Tetraventricular hydrocephalus most likely resulted from impaired CSF resorption caused by protein-rich inflammatory exudate [13]. Its early onset is a recognized marker of poor prognosis [4]. The thoracic spinal cord ischemia may have resulted from several converging mechanisms [5,14]: inflammation of the radiculomedullary arteries within the subarachnoid space, a systemic inflammatory or hypercoagulable state suggested by hyperfibrinogenemia (9.02 g/L), and episodes of septic hypotension compromising a watershed territory with limited collateral circulation. Although the absence of spinal cord compression is a required diagnostic feature and supports an ischemic cause, it does not independently confirm spinal cord infarction [15]. The spinal MRI abnormalities may reflect related consequences of a single thoracic spinal cord lesion rather than three independent lesions. Under this interpretation, thoracic spinal cord atrophy represents the sequela of the infarction, whereas the cervical Wallerian degeneration and lumbar paraspinal muscle atrophy represent possible rostral and caudal consequences, respectively. Polyuria accompanied by hypernatremia and a response to desmopressin suggested central diabetes insipidus, possibly resulting from hypothalamic-pituitary involvement, a complication rarely reported in adults with bacterial meningitis [16]. However, because plasma and urine osmolalities were not measured, the diagnosis remained presumptive. The cause of the flaccid tetraparesis was also uncertain. The absence of documented albuminocytologic dissociation and negative anti-ganglioside results do not exclude an immune-mediated post-infectious polyradiculoneuropathy [7,17,18]. However, recurrent sepsis, prolonged immobilization, and corticosteroid exposure are also risk factors for ICU-acquired weakness. Together with the electrophysiological evidence of diffuse axonal neuropathy, these factors make critical illness polyneuropathy an equally plausible diagnosis [19]. Overall, these four complications likely reflected distinct mechanisms: impaired CSF resorption, vascular inflammation and procoagulant activation, hypothalamic-pituitary dysfunction, and either post-infectious autoimmunity or ICU-acquired neuromuscular weakness. Their coexistence suggests multiple injury processes associated with both the initial central nervous system infection and the subsequent critical illness, rather than a single pathophysiological cascade.

Previous case reports provide useful comparisons. Palasamudram Kumaran et al. described spinal cord infarction developing approximately two weeks into pneumococcal meningoencephalitis despite antibiotic treatment, followed by partial functional recovery over subsequent months. Similarly, our patient developed severe neurological deficits despite improvement in the initial CSF findings. However, no pathogen was identified in our case, and the delayed MRI findings suggested previous ischemic injury rather than documenting an acute infarction. Ding et al. reported Guillain-Barré syndrome with limb symptoms beginning on day 17 following bacterial meningitis and emphasized the differential diagnosis of critical illness polyneuropathy. In our patient, areflexic tetraparesis and diffuse axonal sensorimotor neuropathy raised a similar diagnostic question, but prolonged critical illness and recurrent sepsis prevented confident attribution to a post-infectious immune-mediated neuropathy. These comparisons highlight both the possibility of neurological deterioration despite treatment and the difficulty of determining its precise mechanism during prolonged intensive care [5,7]. 

Multidrug-resistant infections and management in a resource-limited setting

The infectious course illustrates the challenges of diagnosing and managing antimicrobial-resistant organisms during prolonged intensive care. A respiratory molecular panel that was negative on ICU day 2 detected high concentrations of *Acinetobacter calcoaceticus-baumannii* complex and *Klebsiella pneumoniae *48 hours later, suggesting rapid nosocomial acquisition or an increase in organism burden to detectable levels [9]. However, molecular detection alone cannot distinguish infection from colonization or definitively establish the timing of acquisition. The resistance findings must also be described precisely. The cultured *K. pneumoniae *isolate was reported to produce an extended-spectrum beta-lactamase and remained susceptible to carbapenems. However, the detection of CTX-M in a polymicrobial respiratory sample cannot, by itself, assign that gene to a specific organism. Similarly, detection of mecA/mecC and MREJ was consistent with methicillin-resistant *Staphylococcus aureus*, but antimicrobial treatment should be interpreted alongside culture results and clinical evidence of infection. Two antimicrobial management decisions warrant particular discussion. First, amphotericin B was administered after fluconazole-susceptible *Candida tropicalis *was isolated from respiratory secretions, based on the high prevalence of fluconazole resistance in the unit. This decision should be interpreted cautiously because isolation of *Candida *from respiratory secretions usually represents colonization and rarely warrants antifungal treatment. Amphotericin B also carries a substantial risk of nephrotoxicity, which is particularly relevant because the patient subsequently developed oliguric renal failure. Second, empiric antimicrobial therapy was broadened to cefepime at ICU admission; however, the organism subsequently isolated demonstrated intermediate susceptibility to cefepime. This discordance illustrates the limitations of empiric therapy in settings with unfavorable local resistance patterns and underscores the importance of obtaining microbiological specimens promptly and reassessing treatment when susceptibility results become available. The immediate cause of death was septic shock associated with *Enterobacter cloacae *bacteremia in the setting of polymicrobial nosocomial infection. Although the neurological complications likely contributed to the patient's prolonged critical illness and vulnerability to infection, the available evidence suggests that nosocomial infection was the principal proximate cause of death. This is consistent with the findings of Fu et al., who identified multidrug-resistant bacterial infection as an independent predictor of increased mortality and prolonged ICU stays [8]. This case therefore highlights two divergent clinical trajectories: continued neurological deterioration despite normalization of the CSF findings and the emergence of multidrug-resistant nosocomial infections after apparent control of the initial meningeal infection. Appropriate initial antimicrobial therapy and rapid improvement in the CSF profile did not prevent these subsequent complications. Continued neurological monitoring, microbiological surveillance, and infection-prevention measures are therefore essential even after the index infection appears to be controlled, particularly in ICUs with a high prevalence of antimicrobial resistance.

Vaccination and prevention

The patient reported no history of pneumococcal or meningococcal vaccination, and no vaccination records were available. The only objective immunization-related finding was an immunoglobulin G (IgG) concentration against the capsular polysaccharide of *Haemophilus influenzae* type b that exceeded the threshold associated with short-term protection described by Peltola et al. [20]. This finding suggests pre-existing immunity but does not establish whether that immunity resulted from vaccination or natural exposure. Together with the negative soluble antigen test, it made invasive *H. influenzae *type b infection less likely but did not definitively exclude it. More broadly, the absence of reliable vaccination records highlights the importance of documenting and improving pneumococcal and meningococcal vaccination coverage among eligible adults in this setting.

Key messages for clinical practice

Three lessons emerge from this case that may apply to other ICUs in resource-limited settings. First, persistent or progressive headache and spinal pain after apparently minor trauma, particularly when accompanied by new neurological findings, should prompt evaluation beyond a musculoskeletal cause, even in the absence of fever or laboratory evidence of systemic inflammation [1,3]. Second, a negative microbiological workup, including Gram staining, culture, soluble antigen testing, and a syndromic molecular panel, does not completely exclude bacterial meningitis. Although negative results reduce the likelihood of the pathogens covered by these tests, clinicians should consider organisms not included in the panel [11], the possible effects of antibiotic exposure before sampling [12], and noninfectious alternative diagnoses [3]. Third, improvement in the initial CSF findings does not preclude subsequent neurological complications or nosocomial infections. Patients requiring prolonged intensive care therefore need continued neurological assessment, rigorous infection-prevention measures, and prompt microbiological testing when a new infection is suspected [1,5,6,8].

## Conclusions

The coexistence of multiple uncommon neurological complications in meningoencephalitis represents an unusual clinical presentation; to our knowledge, the combination observed here has not previously been reported. A negative microbiological workup does not completely exclude meningoencephalitis when clinical and CSF findings support the diagnosis, but should prompt consideration of alternative infectious and noninfectious etiologies. Improvement in CSF findings does not preclude subsequent neurological deterioration or nosocomial infections. Continued neurological assessment, rigorous infection-prevention measures, and prompt investigation of suspected infections remain essential throughout prolonged intensive care, particularly in resource-limited settings.
